# Total fungal RNA extraction from complex solid- state fermentation products

**DOI:** 10.1007/s00253-026-13946-3

**Published:** 2026-07-06

**Authors:** Mikael Terp, Christian Enrico Rusbjerg-Weberskov, Teis E. Sondergaard, Mette Lübeck

**Affiliations:** Department of Chemistry and Bioscience, Aalborg University, Aalborg, Denmark

**Keywords:** RNA extraction, Filamentous fungi, Solid-state fermentation, Brewer’s spent grain, *Neurospora intermedia*

## Abstract

**Abstract:**

Investigating fungal gene expression in complex solid-state fermentation (SSF) systems is essential for process optimization but is hindered by challenges in RNA extraction. RNA extraction from fungal samples rich in plant biomass is a special challenge that is not met by the commercial RNA extraction kits and conventional RNA extraction methods we have tested. This study presents an optimized RNA extraction method, suitable for RT-qPCR from agricultural side streams fermented by filamentous fungi. The method was applied to investigate the temporal expression of the class II hydrophobin NC2 in *Neurospora intermedia* grown on four different sources of brewer’s spent grain (BSG): IPA, Pilsner, Stout, and a microbrewery batch. Relative NC2 expression showed a clear temporal increase, peaking on day 6, with the microbrewery BSG batch having the highest peak expression. It demonstrates the applicability of the presented RNA extraction method to monitor expression of a gene of interest during solid-state fermentation of a complex substrate. Furthermore, the RNA extraction protocol was successfully validated on other challenging substrates, including rapeseed press cake and oat hulls, and with other fungi (*Aspergillus oryzae* and *Trichoderma asperellum*), consistently yielding high-purity RNA. This work provides a reliable method for RNA extraction from complex SSF products that can be used for gene expression analysis.

**Key points:**

• *Reliable RNA extraction protocol developed for complex fungal SSF substrates*

• *Hydrophobin NC2 expression in N. intermedia might be temporally regulated on BSG*

• *Peak NC2 expression (day 6) was observed on BSG from a microbrewery*

**Supplementary Information:**

The online version contains supplementary material available at https://doi.org/10.1007/s00253-026-13946-3.

## Introduction

Biological side-stream valorization is an important goal for achieving a circular bioeconomy. Within this field, microbial valorization is a highly promising candidate for the upcycling of agro-industrial and food side streams (Nadar et al. 2024). Specifically, filamentous fungi are adept at this process as they can utilize most, if not all, biological side streams (Khatami et al. 2026; Lübeck and Lübeck 2022), and can be grown using solid-state fermentation (SSF), which is a cost-effective alternative to submerged fermentation (Borkertas et al. 2025; Wang et al. 2023).

While filamentous fungi can ferment many biological side streams, the focus of this study is primarily Brewer’s Spent Grain (BSG), with secondary investigations on rapeseed press cake (RPC) and oat hulls (OH). BSG is the solid biological substrate leftover from the mashing process in beer production (Virdi et al. 2025). With an annual beer production of approximately 180 million hL, an estimated 36 million tonnesof BSG is produced annually (Virdi et al. 2025). BSG is mainly used for ruminant feed, biogas production, or disposed of in landfills (Bianco et al. 2020). BSG from industrial beer production generally has a low concentration of starch and is mainly composed of fiber and protein, whereas BSG from microbreweries often retains a higher concentration of starch (Osei et al. 2025). Both sources have a high moisture content, which can decrease their applicability in valorization due to costly preprocessing requirements, i.e., drying or sterilization (Bianco et al. 2020). RPC is the side stream from rapeseed oil production; after pressing, approximately 50% of the initial weight remains as RPC (Wongsirichot et al. 2024). This side stream is high in protein and, depending on the processing method, residual oil (Östbring et al. 2019). Oat hulls are a highly fibrous, low-protein side stream from the dehulling of oats (Schmitz et al. 2020).

Upcycling plant-based side streams with fungal SSF can increase the overall protein concentration (Cooray and Chen 2018; Mohamed Korayem et al. 2025; Radulescu et al. 2025; Rusbjerg-Weberskov et al. 2025). While this can increase the value of a side stream, many cannot be used directly as food/feed, either due to government regulation or the presence of secondary metabolites, such as glucosinolates in RPC (Tie et al. 2020) or mycotoxins like Fumonisins in BSG (Gonzalez Pereyra et al. 2011; Mastanjević et al. 2019; Piacentini et al. 2015). An alternative valorization strategy is the production of specific high-value compounds, such as proteins, which can be selectively extracted, bypassing these limitations.

SSF with *Neurospora intermedia* has been performed on a myriad of side streams, most notably on Okara (a side stream from soymilk production) to produce red Oncom (Gmoser et al. 2020; Maini Rekdal et al. 2023, 2024; Shapiro and Cumbelich 2025; Xiros et al. 2008). *N. crassa/N. intermedia* is known to lack the biosynthetic gene clusters that produce mycotoxins (Bartholomai et al. 2022; Perkins and Davis 2000), and *N. intermedia* has very limited incidence of human infections, with only one case described (Wu et al. 2024) compared to *Rhizopus* spp. (Pham et al. 2023)*.* This safety profile makes *N. intermedia* a compelling choice for side-stream valorization.

High-value proteins like hydrophobins have many applications (Linder et al. 2005; Rojas-Osnaya et al. 2024), but are notoriously difficult to extract from fungal cultures. This can be due to either very low expression levels in wild-type strains (Vereman et al. 2019) or the inherently stable nature of hydrophobins integrated in the cell wall themselves (Linder et al. 2005). For *Neurospora* spp., only the class I hydrophobin EAS has been extracted directly from the fungus (Templeton et al. 1995), whereas the class II hydrophobin NC2 has only been produced recombinantly in *E. coli* (Ren et al. 2014) or in a cell-free expression system (Siddiquee et al. 2020).

Extracting hydrophobins is further complicated by the complex, heterogeneous nature of SSF substrates. Therefore, investigating gene expression via RT-qPCR offers a reliable method for process monitoring and optimization. This approach, however, introduces its own challenge: the efficient and reliable extraction of high-quality RNA from such complex matrices. CsCl ultracentrifugation has been effectively applied for RNA extraction from fungi grown on complex lignocellulosic substrates, enabling the recovery of high-quality RNA from challenging matrices (Mäkelä and Hildén, 2018; Patyshakuliyeva et al. 2014). Alternatively, TRIzol followed by alcohol precipitation or column purification has been widely utilized, mainly for pure mycelial samples (Bernáldez et al. 2017; Cortés-Maldonado et al. 2020; Schumann et al. 2013). Column purification steps or additional clean-up procedures are often necessary when using TRIzol, especially with samples containing large amounts of polysaccharides or solid substrates (Cortés-Maldonado et al. 2020; Schumann et al. 2013). While CsCl ultracentrifugation remains a promising method for obtaining high-integrity RNA from fungi grown on lignocellulosic materials, it requires access to specialized ultracentrifuge equipment, which may not be available in all laboratories (Mäkelä and Hildén 2018; Patyshakuliyeva et al. 2014). Several commercial RNA extraction and purification kits have also been tested for fungal RNA extraction, showing variable results depending on the fungal species and substrate composition (Chauhan et al. 2018). Unlike animal cells, filamentous fungal cell walls are highly recalcitrant to mechanical and chemical disruption due to their complex composition of chitin, β-glucans, and mannoproteins. Effective RNA extraction necessitates vigorous mechanical lysis combined with strong chaotropic agents to release nucleic acids while mitigating coprecipitation with structural polysaccharides and rapid degradation by endogenous RNases (Ost et al. 2025; Siniecka-Kotula et al. 2024).

To the best of our knowledge, no RNA extraction method has been specifically validated for handling complex SSF substrates with subsequent quantification of fungal gene expression.

**Fig. 1:**
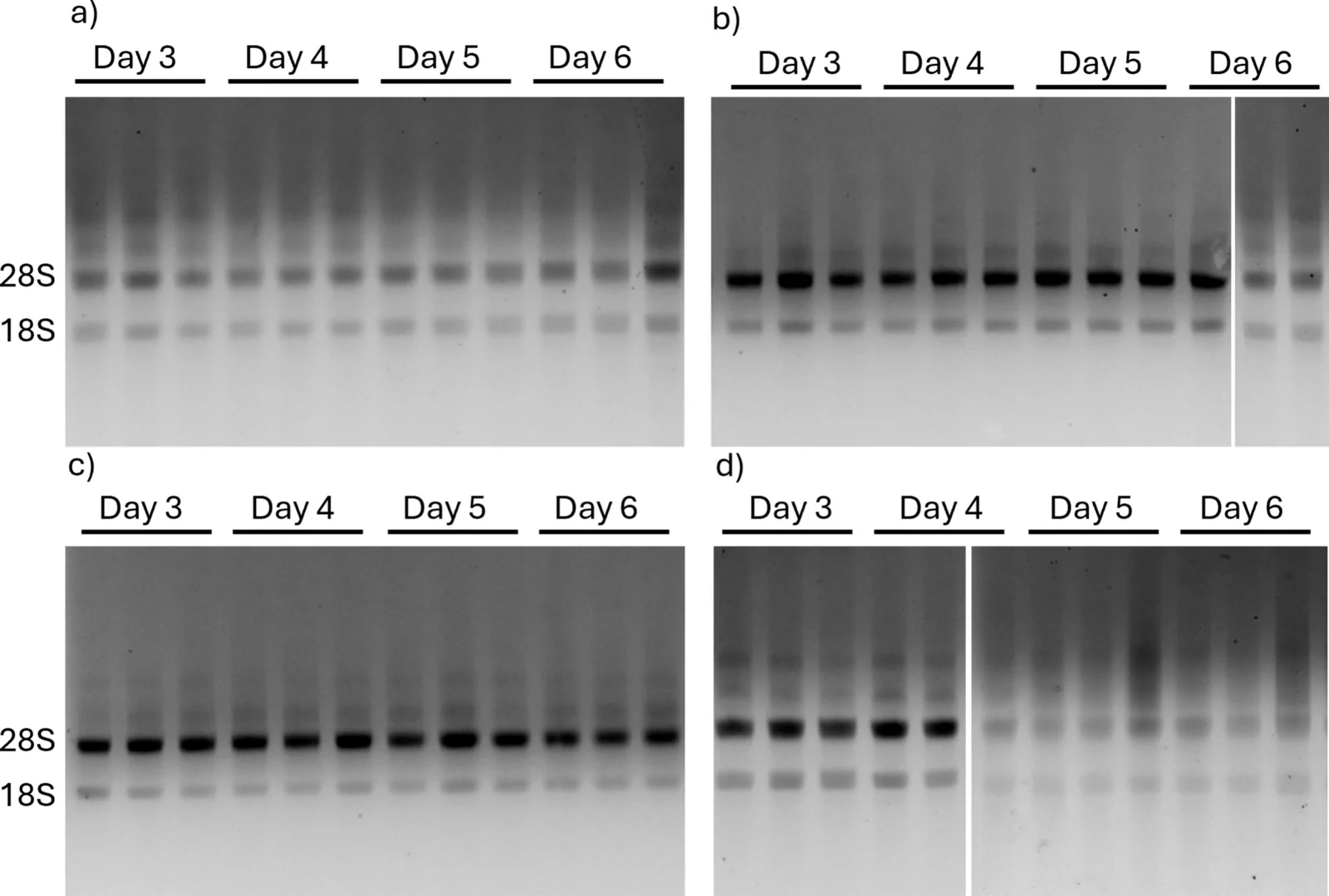
RNA extraction visualised on a 1% agarose, 1% bleach gel. With DNA smears above the 28S and 18S bands for all samples. BSG-A (**a**), BSG-B (**b**), BSG-C (**c**), BSG-D (**d**), with bars showing the days for the biological triplicates

In this study, we present an RNA extraction method optimized specifically for complex SSF matrices, such as fiber- and protein-rich BSG, that utilizes a cheap RNA buffer for storage and handling of the substrates. The resulting RNA is of high quality and can be used to investigate gene expression in complex SSF samples. We applied this method to investigate the temporal expression of the hydrophobin NC2 homolog (Supplementary 1 Fig. 1) from *N. intermedia* NRRL 2884 in SSF of several BSG sources using RT-qPCR. We also tested the broader applicability of the RNA extraction method on OH and RPC substrates with two additional fungi: *Trichoderma asperellum* TA1 and *Aspergillus oryzae* CBS 466.91.

## Materials and methods

### Fungal strain

*Neurospora intermedia* NRRL 2884 (Terp et al. 2025) was acquired from ATCC® (36935™), originally isolated from Oncom, Indonesia. Stored at −80 °C as conidia in 1 M Sorbitol (Fisher Scientific, Roskilde, Denmark). *N. intermedia* NRRL 2884 was inoculated onto Vogel medium N (Metzenberg 2003), 2% agar (VWR, Søborg, Denmark), incubated for 1 week at 35 °C. Mycelia and conidia were scraped off agar plates added to autoclaved water and vortexed before being filtered through double-layered sterile Miracloth (Merck Millipore, Søborg, Denmark). The conidia were then counted in a hemacytometer and used immediately for SSF.

###  Solid-state fermentation of *Neurospora intermedia* on brewer’s spent grain

To test if different sources of BSG had an influence on RNA extraction and the expression of NC2, four different types of BSG flour were included in the study. Three of these were commercially available from the company Agrain (Copenhagen, Denmark) and had been used in the production of Indian pale ale (IPA) (BSG-A), Pilsner (BSG-B), and Stout (BSG-C) and were supplied as pre-dried, uniform flours. The fourth BSG sample (BSG-D) was obtained from the microbrewery Slowburn (Albertslund, Denmark) and processed in-house. The BSG from Slowburn was oven-dried at 60 °C for 24 h, blended in a Thermomixer TM31 (Vorwerk, Wuppertal, Germany) for 15 s, followed by milling (Laboratory Mill 120, PerkinElmer, MA, USA) through a 500-µm mesh. Proximate composition of the four BSG samples is shown on Table 1.

**Table 1:** Proximate composition of BSG sources BSG-A (IPA), BSG-B (Pilsner), BSG-C (Stout), BSG-D (Microbrewery BSG). Values are given per 100 g of dry matter (d.m.)

Constituent	BSG-A*	BSG-B*	BSG-C*	BSG-D**
Energy (kJ/kcal)	1205/292	1186/287	1205/292	1367/328
Protein (% d.m.)	18.4	17.6	19.4	16.8
Dietary fibers (% d.m.)	58.2	57.5	59.0	42.93 ± 1.01
Carbohydrates (% d.m.)	7.0	9.7	6.1	29.3
Sugars (% d.m.)	1.0	2.2	0.9	3.2
Total fat (% d.m.)	8.3	7.1	8.1	6.5
Saturated fat (% d.m.)	2.1	1.8	2.1	1.7
Sodium (mg/100 g d.m.)	80	80	127	30.6

^*^Proximate composition of BSG-A, BSG-B and BSG-C from Agrain’s data sheet

^**^Proximate composition of BSG-D (Rusbjerg-Weberskov et al. 2025)

Water was added to BSG to a moisture content of 70% and autoclaved at 121 °C for 40 min. The loss of water from autoclaving was measured and sterile water was added to adjust water content back to 70%. The prepared BSG was inoculated with *N. intermedia* NRRL 2884 to a final concentration of 3.6 ∙10^5^ conidia/g dry weight. The inoculated media was aliquoted in biological triplicates for the fermentation days of 3, 4, 5, and 6 days to petri dishes (Ø = 30 mm) (VWR, Søborg, Denmark) with 6 ± 0.1 g of wet weight and was incubated at 35 °C. Triplicate samples were taken out for each respective day and immediately frozen in liquid N_2_ before being lyophilized (Scanvac Coolsafe Freeze Dryer, LaboGene, Lillerød, Denmark).

###  RNA extraction from brewer’s spent grain

After samples were lyophilized, they were ground to a fine powder (in liquid N_2_) with a mortar and pestle, subsequently resuspended in RNA buffer (25 mM Sodium Citrate, 10 mM EDTA, 0.189 M ammonium sulphate, pH 5.2. VWR, Søborg, Denmark) (Malmstrom 2015) This buffer formulation utilizes EDTA and Sodium Citrate for the chelation of divalent and trivalent -cations, thereby inhibiting Mg-dependent RNases, while ammonium sulphate further restricts RNase activity and stabilize RNA structural integrity (Chheda et al. 2024). Samples were resuspended n a ratio of 10 mL RNA buffer to 1.8 g dried sample (10 mL buffer per sample), vortexed for 5 s and stored at −80 °C until use. On the day of extraction, samples were thawed and kept on ice.

The samples were vortexed, then 250 µL sample was pipetted with a cut pipette tip to a sterilized 1.5 mL tube and centrifuged for 5 min at 14,100 RCF (Eppendorf MiniSpin, Fisher Scientific, Roskilde, Denmark). The RNA buffer was decanted, then the samples were mixed with 1 mL TRIzol (Thermo Fisher Scientific, Roskilde, Denmark) and 13.75 µL glycogen solution (18 g/L Glycogen, RNA grade (Thermo Fisher Scientific, Roskilde, Denmark) in RNA Buffer), and vortexed for 30 s at 3000 RPM (IKA MS2 minishaker, Sigma-Aldrich, Søborg, Denmark). The samples were centrifuged for 5 min at 14,100 RCF and the supernatant was transferred to new 1.5 mL tubes by decanting if no particulates were in the supernatant, otherwise they were carefully pipetted to avoid particulates. Subsequently 0.2 mL chloroform (VWR, Søborg, Denmark) was added; the samples were then vortexed for 30 s at 3000 RPM and centrifuged for 15 min at 14,100 RCF. Afterwards 500 µL supernatant was transferred to a new tube and mixed in a 1:1 ratio with 70% ethanol; 700 µL of this solution was transferred to a RNeasy minispin (Qiagen, Aarhus, Denmark) column and the manufacturer’s protocol was followed for RNA purification; elution volume was 30 µL for each sample.

RNA concentration and purity were assessed on a NanoDrop One (Fisher Scientific, Roskilde, Denmark), and subsequently RNA concentration was adjusted to 143 ng/µL, and stored at −80 °C, for subsequent use the next day.

RNA integrity was assessed on a 1% Agarose (VWR, Søborg, Denmark), 1% household bleach (Salling Klor, Aalborg, Denmark) gel (Aranda et al. 2012). Here, 1 µg RNA of each sample was mixed with 6 × purple loading dye (New England BioLabs, Herlev, Denmark) and run in a RunOne EP-2100 (EmbiTec, San Diego, Denmark) at 100 v for 30 min stained with ethidium bromide (Fluka, Fisher Scientific, Roskilde, Denmark).

###  RT-qPCR

cDNA was synthesized with Oligo dT_18_ primers using the RevertAid First Strand cDNA Synthesis Kit (Fisher Scientific, Roskilde, Denmark). Oligo dT18 primers were utilized to specifically target polyadenylated mRNA, ensuring the selective amplification of transcriptionally active genes and minimizing ribosomal RNA background amplification. DNA was degraded with DNase I, RNase-free (Fisher Scientific, Roskilde, Denmark) and the manufacturer’s protocol was followed for synthesis and DNase treatment.

Primers for RT-qPCR were designed manually on the genome of *N. intermedia* NRRL 2884 (Terp et al. 2025) and were designed to span from one exon to the next. The target gene was NC2, the class II hydrophobin (NCU08192, *Neurospora crassa* OR74A) (Forward: 5′ ACTCCCTGCTCCGGCCTCT 3′, Reverse: 5′ CGATGGCAGCGCAGGTAGACT 3′), and the reference gene Beta-tubulin (NCU04054, *Neurospora crassa* OR74A) (Forward: 5′ CGCAACGGTCGTTACCTCACCT 3′, Reverse: 5′ AGGGCAGTCTGGACGTTGTTGG 3′) (Cusick et al. 2014). Primers were ordered from Eurofins Genomics (Cologne, Germany).

RT-qPCR reaction was performed in technical triplicates for each sample using the Brilliant III Ultra-Fast SYBR Green High ROX (Agilent, Glostrup, Denmark). RT-qPCR was done following the manufacturer’s protocol except the working volume was adjusted from 20 to 10 µL with a final primer concentration of 100 nM of each primer. qPCR was run on a Stratagene Mx3005P (Agilent Technologies, Glostrup, Denmark) with Rox using the method of 3 min 95 °C, 40 cycles of 95 °C for 10 s, 60 °C for 20 s then 1 cycle of 95 °C for 1 min, 55 °C for 30 s, 95 °C for 30 s.

#### Fungal strains – *Aspergillus oryzae* and *Trichoderma asperellum*

To test if the RNA extraction method was usable on other side streams and with other fungi, two extra fungi were included.

*Aspergillus oryzae* CBS 466.91 was acquired from the WI-KNAW culture collection in the Netherlands, which was originally isolated in Osaka, Japan. *Trichoderma asperellum* TA1 was isolated from field soil at a farm belonging to the University of Copenhagen, Denmark (Terp et al. 2025). All isolates were stored at −80 °C either as agar plugs or spores in 15% glycerol (Merck Millipore, Søborg, Denmark).

To isolate spores, *A. oryzae* CBS 466.91 and *T. asperellum* TA1 were grown on yeast extract peptone glucose (YPG) agar plates (Glucose 25 g/L, Peptone 20 g/L, Yeast extract 10 g/L, Agar 20 g/L, Fisher Scientific, Roskilde, Denmark) at 30 °C for a week. Mycelia and spores were scraped off agar plates added to autoclaved water and vortexed before being filtered through double-layered sterile Miracloth. The spores were then counted in a hemacytometer and used immediately.

#### Solid state fermentation of *Aspergillus oryzae*, *Trichoderma asperellum*, and *Neurospora intermedia *on brewer’s spent grain, oat hulls, and rapeseed press cake

With the two additional fungi added we also wanted to add two extra side streams and chose oat hulls (OH) and rapeseed press cake (RPC).

Milled organic OH were supplied by Good Food Group (Vejle, Denmark). Milled double-low RPC was supplied by DLG Food Oil (Dronninglund, Denmark) where the *Brassica napus* was cleaned and conditioned twice at 100 °C before pressing and the resulting RPC was stored at 5 °C. The supplied RPC was sieved through a fine mesh sieve 500 µm (Endecotts, London, UK), before fermentation to remove large particulates. Pilsner BSG was the same as described in the “Solid-state fermentation of Neurospora intermedia on brewer’s spent grain” section, i.e., BSG-B. Proximate composition of RPC was supplied by DLG Food Oil, Proximate composition of milled organic OH was supplied by Good Food Group (Table 2). Fermentation and RNA extraction were performed as was described in the sections.

**Table 2:** Proximate composition of oat hulls (OH), rapeseed press cake (RPC)

Constituent	OH	RPC
Energy (kJ/kcal)	967/231*	1234/295*
Protein (% d.m.)	5.3	29.5
Dietary fibers (% d.m.)	68	12
Carbohydrates (% d.m.)	14.2	2.4
Sugars (% d.m.)	< 1	10
Total fat (% d.m.)	1.9	11.5
Saturated fat (% d.m.)	0.5	n/a
Sodium (mg/100 g d.m.)	135	10

^*^Estimated based on macronutrients

### Data analysis

RNA extraction and RT-qPCR data were filtered and analysed with custom python (v3.12.6) scripts, using the packages: SciPy (v1.14.1) (Virtanen et al. 2020), Pandas (v2.2.2) (pandas development team, 2025), Matplotlib (v3.9.2) (The Matplotlib Development Team 2025), Seaborn (v0.13.2) (Waskom 2021), Scikit-Posthocs (v0.9.0) (Terpilowski 2019), and StatAnnotations (v0.7.2) (Charlier et al. 2023). The script and datasets were made in an interactive Python notebook format for ease of use and are available at https://github.com/TerpmikaelAAU/Temporal_NC2.

RNA extraction results were tested for normality with the Shapiro–Wilk test. Kruskal–Wallis *H* test was performed, and if *p* < 0.05, the Posthoc Dunns test was run. RT-qPCR results were also checked for normality with the Shapiro–Wilk test. Relative expression of NC2 between days of fermentation was calculated via the ${2}^{-\Delta \Delta {C}_{t}}$ method (Livak and Schmittgen 2001) with Beta-tubulin as the reference, and day 3 expression as ${t}_{0}$. RT-qPCR data were plotted with Seaborn and statistically tested with the Mann–Whitney *U* Test, with Bonferroni correction using StatAnnotations. Calculation of relative expression levels between substrates, was done using IPA expression as the target reference instead of day 3 expression.

## Results

### RNA purity evaluation

The four BSG types (IPA: BSG-A, Pilsner: BSG-B, Stout: BSG-C, Microbrewery BSG: BSG-D) were fermented at 35 °C for 3, 4, 5, and 6 days in biological triplicates. RNA was successfully extracted from all BSG types, and the RNA quality and integrity was assessed to ensure suitability for downstream RT-qPCR application to determine the temporal expression of NC2. Preliminary extraction trials were conducted (Supplementary files 1, Table S1).

Concentration and purity of RNA samples were determined with spectrophotometry (Table 3). The mean 260/280 was 2.23 ± 0.02 and the 260/230 ratio was 2.52 ± 0.15. Pure RNA has a 260/280 and 260/230 ratio of 1.8 or above (Desjardins and Conklin 2010), with ratios below 1.8 indicating contaminants, e.g, phenol or chloroform. Thus, there was no carryover of extraction/purification solvents with this method. The RNA concentration was 991.35 ± 192.80 ng/µL across the four types of BSG substrate and the total RNA yield ranged from around 15 µg to 18 µg per gram of dry matter (DM) sample. According to the Kruskal–Wallis *H* test there was no difference between RNA purity ratios and RNA concentration when comparing between fermentation times (*p* > 0.05). No difference was found between RNA purity ratios when comparing substrates (*p* > 0.05). When comparing RNA concentration between substrates with the Kruskal–Wallis *H* test showed a significant difference (*p* < 0.05), after the Bonferroni correction for 6 pairwise comparisons Dunn’s test showed a significant difference between BSG-A and BSG-B (*p* = 0.042).

**Table 3:** RNA purity and concentration of *N. intermedia* extracted from BSG-A, BSG-B, BSG-C, and BSG-D fermented for 3, 4, 5, and 6 days. Kruskal–Wallis H-test showed a statistically significant difference between RNA concentration when comparing substrates (*p* = 0.035). After Bonferroni correction for 6 pairwise comparisons Dunn’s test showed a statistically significant difference between BSG-A and BSG-B (*p* = 0.042)

Substrate (*n* = 12, *N* = 48)	260/280	260/230	Concentration [ng/µL]	Total RNA yield [µg/DM]*
BSG-A	2.23 ± 0.01	2.54 ± 0.08	1016.75 ± 257.16	18.36 ± 3.25
BSG-B	2.23 ± 0.01	2.51 ± 0.09	861.71 ± 108.38	14.75 ± 1.80
BSG-C	2.24 ± 0.01	2.52 ± 0.13	944.45 ± 182.68	15.82 ± 3.08
BSG-D	2.23 ± 0.02	2.47 ± 0.24	1040.82 ± 242.40	17.16 ± 3.56

^*^Estimated values based on input dry matter (DM)

This shows that the RNA extraction method resulted in consistent purity, and a slight variation in RNA concentration between BSG-A and BSG-B.

#### RNA integrity evaluation

The RNA integrity was assessed by bleach gel electrophoresis and shows clear 28S rRNA and 18S rRNA bands (Fig. 1a–d), which is indicative of high RNA integrity (Skrypina 2003; Taylor et al. 2010). Slight smearing was observed in some samples, but most were above the ribosomal RNA bands and were fragmented genomic DNA as the bands and smears are located above the 28 s rRNA bands. This underlines the importance of including DNase treatment in this RNA extraction method.

#### Temporal expression of NC2 in brewer’s spent grain

High RNA quality was confirmed by spectrophotometry and electrophoresis. Next, its applicability in an RT-qPCR experiment was investigated.

The RT-qPCR experiment was performed on all the samples in technical triplicates. The reference gene of Beta-tubulin had a comparable expression level in all samples determined by a similar Ct value with a standard deviation < 1 across all samples (Supplementary 2, Table S1-S2). For the technical replicates, the Ct values of both Beta-tubulin and NC2 had a standard deviation of < 0.4 (Supplementary 2, Table S3, S5), and the dissociation curves were consistent across all samples (Supplementary 1, S2-6). These results demonstrate a robust RT-qPCR experiment.

For the gene of interest, NC2, the Ct value also displayed a standard deviation below 1 within the biological replicates for each time point in the fermentation (Supplementary 2, Table S4), which demonstrates that the RNA extraction method is highly reproducible alongside the fermentation.

**Fig. 2:**
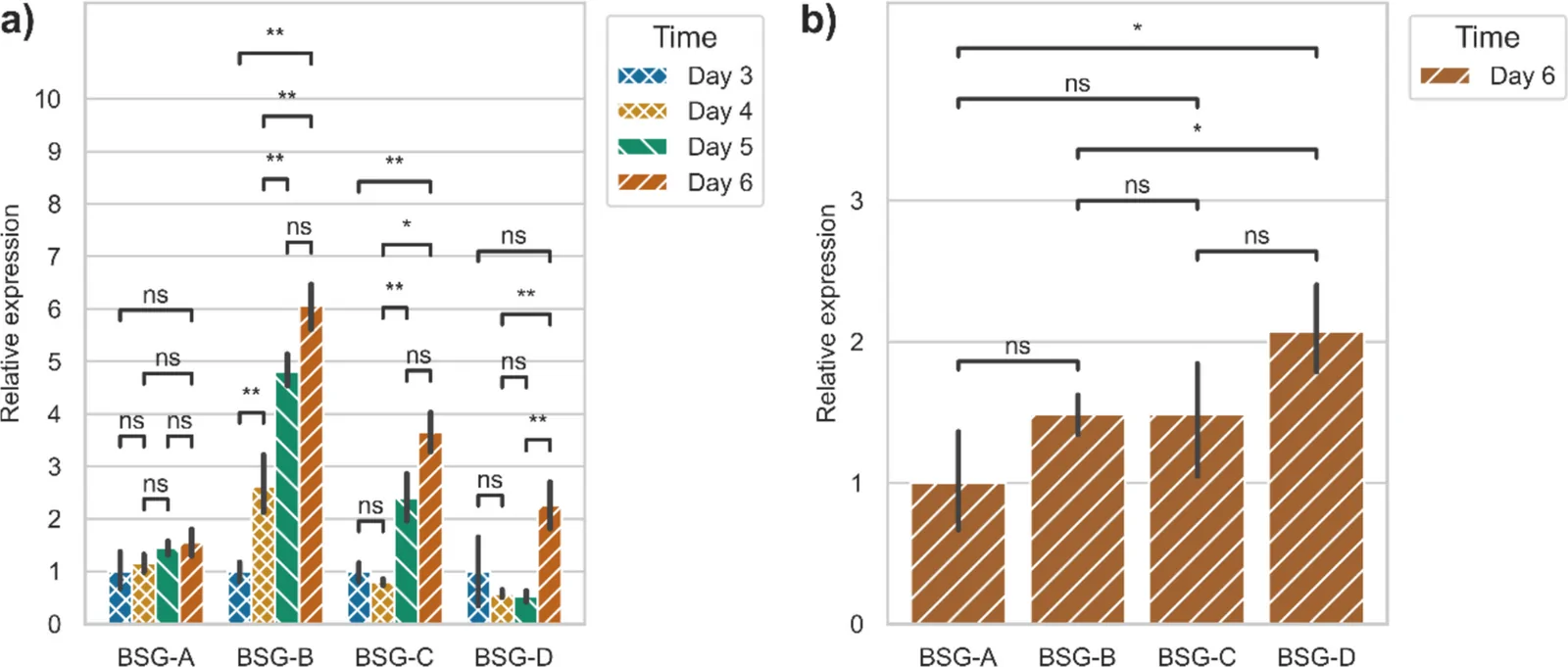
Bar plot of temporal relative expression of NC2 hydrophobin in different sources of BSG, with day 3 expression within each substrate as the reference expression, temporal relative expression within each individual substrate was statistically evaluated between distinct time points with the Mann–Whitney U test (**a**). Bar plot of relative expression of NC2 in different sources of BSG at day 6 with BSG-A as the reference expression, temporal relative expression between day 6 expression in each individual substrate was statistically evaluated with the Mann–Whitney U test (**b**). ns: 0.05 < *p* ≤ 1, *: 0.01 < *p* ≤ 0.05, **: 0.001 < *p* ≤ 0.01

The temporal relative expression levels of NC2 in the different BSG sources were calculated and are presented in Fig. 2a. Differences in expression level of NC2 were analysed with Mann–Whitney *U* test. In BSG-A, there was no change in relative expression. In BSG-B and BSG-C, there was an increase in expression until day 5 where it reached a plateau. In BSG-D, NC2 expression decreased from day 3 to 4 and increased again on day 6. The experiment showed a temporal difference in NC2 expression with slight variations between substrates.

With the BSG-A samples being statistically identical, they were used as a reference in a comparison between substrates. This would give an indication if there was an actual difference in expression between the substrates and, as shown on Fig. 2b, there was a significant higher difference between BSG-D compared to BSG-A and BSG-B on fermentation day 6. The relative expression of BSG-B on day 3 was also significantly lower than BSG-A and BSG-D on day 3 (*p* < 0.05). The case study of screening temporal NC2 expression on cultivation of different types of BSG identified BSG-D as the substrate that provides highest NC2 expression on day 6 and illustrates the applicability of the presented RNA extraction methods.

### Additional testing of RNA extraction method

To assess the universality of the proposed RNA extraction method, we applied it to various substrates fermented with different fungal species. BSG-B, milled OH, and milled RPC served as the substrates, and *Aspergillus oryzae* and *Trichoderma asperellum* were included alongside *N. intermedia*. All three fungi visibly colonized their respective substrates after 4 days of SSF at 35 °C.

The RNA concentration varied between substrates for *A. oryzae* and *T. asperellum* compared to *N. intermedia* (Table 4); however, with concentrations ranging from 200 to 800 ng/µL and yields ranging from 3.22 to 14.52 µg/DM; this is still more than enough for most RNA-based applications. The 260/280 and 260/230 purity revealed high RNA quality with values above 1.8 for all samples. Kruskal–Wallis *H*-test showed no difference in RNA concentration when comparing between fungi (*p* > 0.05), however, a statistically significant difference when comparing RNA concentration grouped by substrates (*p* = 0.042) was observed; however, after the Bonferroni correction for 3 pairwise comparisons the post hoc Dunn’s test showed no difference (*p* > 0.05); this is due to the loss of power from the Bonferroni correction (VanderWeele and Mathur 2019). Nonetheless, as seen in Table 4 there was a large range of RNA concentrations (min 174 ng/µL, max 1215 ng/µL), which is the main impact on an experimental setting as a too low RNA yield will limit the application of the samples. The post hoc Dunn’s test showed a difference between *N. intermedia* and *T. asperellum* when comparing 260/280 ratio grouped by fungi (*p* = 0.037). However, *N. intermedia *and* T. asperellum* had 260/280 ratios of a median (IQR) = 2.22 (2.20–2.24) and median (IQR) = 2.20 (2.18–2.21), respectively, which in RNA-based application is an insignificant difference.

**Table 4:** RNA concentration and purity for OH, BSG-B, and RPC fermented with *A. oryzae*, *N. intermedia*, and *T. asperellum*. Kruskal–Wallis H-test showed a statistically significant difference when comparing RNA concentration grouped by substrates (p = 0.042), however after Bonferroni correction for 3 pairwise comparisons the post hoc Dunn’s test showed no difference (*p* > 0.05)

Fungi (n = 3, N = 27)	Media	260/280	260/230	Concentration [ng/µL]	Total RNA Yield [µg/DM]*
*A. oryzae*	OH	2.19 ± 0.02	2.28 ± 0.39	221.17 ± 28.14	3.69 ± 0.47
*A. oryzae*	BSG-B	2.21 ± 0.01	2.40 ± 0.19	757.23 ± 41.73	12.62 ± 0.70
*A. oryzae*	RPC	2.22 ± 0.01	2.25 ± 0.50	871.07 ± 446.41	14.52 ± 7.44
*N. intermedia*	OH	2.21 ± 0.01	2.44 ± 0.14	639.17 ± 81.46	10.65 ± 1.36
*N. intermedia*	BSG-B	2.25 ± 0.01	2.51 ± 0.11	439.83 ± 48.64	7.33 ± 0.81
*N. intermedia*	RPC	2.21 ± 0.02	2.42 ± 0.08	314.20 ± 143.52	5.24 ± 2.39
*T. asperellum*	OH	2.21 ± 0.01	2.18 ± 0.27	268.77 ± 68.70	4.48 ± 1.15
*T. asperellum*	BSG-B	2.20 ± 0.01	2.48 ± 0.05	729.83 ± 34.78	12.16 ± 0.58
*T. asperellum*	RPC	2.18 ± 0.02	2.32 ± 0.09	192.93 ± 25.97	3.22 ± 0.43

^*^ Estimated values based on input dry matter (DM)

This demonstrates that RNA purity remained consistent across the three fungal species and substrates, whereas the RNA yield showed greater variability.

**Fig. 3:**
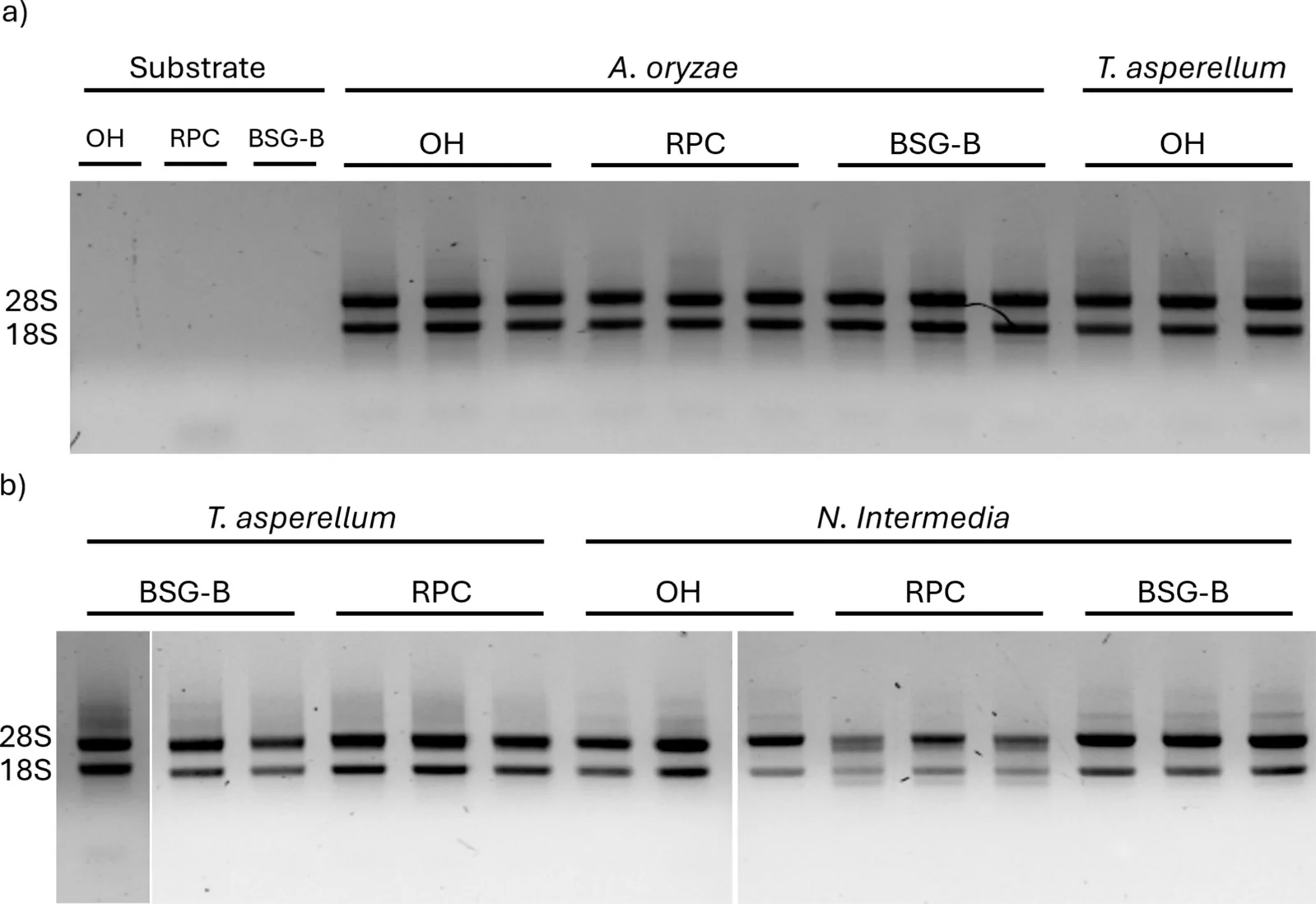
RNA extraction visualised on a 1% agarose, 1% bleach gel. The raw side streams after autoclaving were extracted as a control and show no RNA bands. All extractions display clear 28S/18S ribosomal RNA bands and a distinct indication of genomic DNA. For RPC, the *N. intermedia* extraction shows either very clear large mRNA bands or signs of slight degradation of both the 28S and 18S band. The bars show the biological triplicates for each substrate and which fungus the extraction was from

The RNA integrity for the additional substrates and fungi showed clear 28S and 18S ribosomal RNA bands (Fig. 3a–-b), which was very promising as these substrates have a large difference in their composition (Tables 1 and 2). A smaller amount of DNA smears above the ribosomal RNA bands was also observed, which makes the intensity for the ribosomal RNA bands clearer as compared to some of the earlier RNA extracts. As an additional control for the RNA extraction of the additional substrates, they were also extracted without fermentation and showed no detectable RNA bands (Fig. 3a), however, a slight band was detectable in the milled RPC, which most likely was degraded DNA (Fig. 3b).

## Discussion

Quantifying gene expression in solid-state fermentation (SSF) systems is essential for process optimization, but it presents significant methodological challenges. This is particularly true for complex, heterogeneous substrates like brewer’s spent grain (BSG) and for proteins like hydrophobins, which are notoriously difficult to quantify via standard protein extraction and SDS-PAGE analysis (Linder et al. 2005). Consequently, RT-qPCR is a preferred method, yet it demands a reliable and efficient RNA extraction protocol. Our initial investigations confirmed that standard RNeasy mini and Trizol failed to consistently yield high-quality RNA from our *Neurospora intermedia* SSF samples (Supplementary 1, Table S 1).

To address this, we developed an optimized protocol specifically tailored for complex, heterogeneous solid-state fermentation matrices, such as fiber- and protein-rich BSG, combining a lab-made RNA buffer for optimized sampling followed by Trizol-based lysis with RNeasy column purification. While this combination of Trizol and RNeasy mini has been reported before (Bernáldez et al. 2017; Cortés-Maldonado et al. 2020; Schumann et al. 2013), its application for quantitative analysis from complex fungal SSF on BSG is not well-established. A key refinement was the integration of a simple, lab-made RNA buffer, which proved more economical than commercial alternatives like RNAlater. This buffer allowed for rapid homogenization and representative sampling of the otherwise dry, heterogeneous substrate. Furthermore, it enabled stable sample storage on ice and streamlined the extraction workflow, which in our hands, could be completed in approximately 1 h per 12 samples post-lyophilization. This method consistently produced high-purity RNA (260/280 ratio ≈ 2.2, 260/230 ≈ 2.5), with RNA and DNA generally accepted to be pure with a 260/280 ratio above 1.8 (Koetsier and Cantor 2019), and our results (Table S1-S5) showed it was suitable for downstream RT-qPCR. The presence of DNA in the extracted RNA samples (Fig. 1a-c, Fig. 3a, b) makes DNase treatment imperative for this method as the co-extracted DNA was detectable in preliminary RT-PCR experiments without DNase treatment (Supplementary 1, S9).

Using this validated protocol, we investigated the temporal expression of the class II hydrophobin NC2 homolog in *N. intermedia* grown on four distinct BSG sources (IPA: BSG-A, Pilsner: BSG-B, Stout: BSG-C, Microbrewery BSG: BSG-D). We observed a clear temporal difference in relative NC2 expression for BSG-B, BSG-C, and BSG-D, with all three showing a significant increase in expression that peaked on day 6 of fermentation (Fig. 2a). In contrast, BSG-A exhibited no significant temporal modulation of NC2 expression across the 6-day period. This lack of temporal change in BSG-A, and the distinct peaks in the others, might suggests that the fermentation process is actively modulated by the substrate source.

We initially hypothesized that these differences in expression might be linked to the proximate composition of the BSG. However, BSG-A, B, and C were compositionally similar, with only BSG-D (sourced from a microbrewery) containing a significantly higher concentration of simple carbohydrates (Table 1). Our initial expectations were that this elevated carbohydrate level in BSG-D would translate into a lower overall expression of NC2 as some hydrophobins are linked to lignocellulosic degradation (Brown et al. 2016; Delmas et al. 2012). However, this was not the case as when comparing the peak expression levels on day 6 across all substrates, we found BSG-D to be the highest (Fig. 2b).

It could also be that the distinct temporal expression peak of NC2 on day 6 across most BSG types might correlate more with residual protein composition or specific fiber architectures resulting from differing mashing temperatures and input substrate for the resulting BSG, rather than the proximate composition. Following that BSG-A had the same proximate composition as BSG-C and D but had a consistent expression instead of an expression that peaked on day 6. Which might also explain why BSG–D had a differing expression as this BSG was pre-processed in-house, and some of the expression is explained by the milling process.

We further evaluated the robustness of our RNA extraction method by applying it to two additional complex substrates, oat hulls (OH) and rapeseed press cake (RPC), using *N. intermedia*,* Trichoderma asperellum* and *Aspergillus oryzae*. While the protocol still yielded high-purity RNA across all combinations of fungus and substrate, the final RNA concentration and total yield displayed a moderate degree of variability. RNA extraction of the non-fermented substrates showed no plant RNA as these substrates are heat treated several times in production and autoclaved for fermentation which breaks down most RNA (Chheda et al. 2024).

This study was conducted in small-scale petri dishes with milled substrates, which allowed for rapid screening and simplified representative sampling. The method validated in this study might be applicable to industrial scale SSF, as it seems to be reliable on diverse complex substrates, and might only require some extra mechanical homogenization before or after buffer integration either with wet milling or dry milling before RNA extraction. As in industrial scale SSF most substrates would not be milled prior to the fermentation (Mattedi et al. 2023; Pallín et al. 2024; Qasim et al. 2025). This extra preprocessing step should not influence the validity of the method but should still be validated in the future.

In our preliminary experiments (Supplementary 1, S1) we found that this method does not work on substrates with a very high pH. Its performance and sensitivity at low fungal biomass concentrations remain undetermined, however with yields of 10 µg/DM this should not be a problem in BSG but might be a limiting factor when for example doing SSF on OH with *A. oryzae* or on RPC with *T. asperellum* as these two combinations had the lowest yield of the tested combinations, therefore the method should in the future also test other combinations of fungi and other heterogeneous agro-industrial side-streams.

## Conclusion

In conclusion, we established an optimized RNA extraction protocol customized for complex SSF matrices, validating its utility through the temporal quantification of NC2 expression in *Neurospora intermedia* grown on diverse BSG types. This methodology facilitates robust gene expression analysis, which is crucial for SSF process optimization, the elucidation of fungal metabolic pathways. Future investigations should prioritize the validation of this protocol on un-milled, industrial-scale SSF samples and its integration with advanced transcriptomic technologies.

## Supplementary Information

Below is the link to the electronic supplementary material.

Supplementary 1: Fig. S1: Clustal O alignment of *Neurospora crassa* OR74A hydrophobin NC2, and *Neurospora intermedia* NRRL 2884 NC2 homolog. Fig. S2-6: Dissociation curves from RT-qPCR. Table S1: Preliminary tested RNA extraction methods and resulting concentration, 260/280 and 260/230 ratios. Fig. S7 and Fig. S8: Preliminary tested RNA extraction methods and resulting RNA integrity. Fig. S9: genomic DNA contamination in RT-PCR before adding DNase treatment (DOCX 698 KB)

Supplementary 2: Excel sheets with statistics of Ct values. Table S1: BT_overall, Table S2: BT_Biological_replicates, Table S3: BT_Technical_replicates, Table S4: NC2_Biological_replicates, Table S5: NC2_Technical_replicates (XLSX 15.0 KB)

## Data Availability

Data generated from this study are available as supplementary data and at GitHub (https://github.com/TerpmikaelAAU/Temporal_NC2), where the python code used to generate the figures and data analysis from the study are available in interactive notebooks.

## Declarations

### Author agreement

All authors have read and approved the final version of the submitted manuscript.
