# Supplementary material for: Total fungal RNA extraction from complex solid- state fermentation products
Source: Appl Microbiol Biotechnol. 2026 Jul 6;110:290. doi: 10.1007/s00253-026-13946-3 (PMC13620088; doi:10.1007/s00253-026-13946-3)
Supplement: Supplementary file 1 [file 253_2026_13946_MOESM1_ESM.docx]

# 1. Alignment

Fig. S 1 - Clustal O alignment of Neurospora crassa NC2 and homolog found in Neurospora intermedia

# 2. Melting Curves From RT-qPCR

All dissociation curves were generated post-amplification to confirm primer specificity. Beta-tubulin (BT) targets consistently exhibited a melting peak at approximately 85 °C, while the NC2 target exhibited a melting peak at approximately 89 °C.

- **Fig. S2** – Dissociation curves for *N. intermedia* cultivated on BSG-A.
- **Fig. S3** – Dissociation curves for *N. intermedia* cultivated on BSG-B.
- **Fig. S4** – Dissociation curves for *N. intermedia* cultivated on BSG-C.
- **Fig. S5** – Dissociation curves for *N. intermedia* cultivated on BSG-D.
- **Fig. S6** – Dissociation curves for control reactions performed on genomic DNA (gDNA) templates. Genomic DNA displayed distinct BT and NC2 melting peaks at 85 °C and 87 °C, respectively, confirming clear discrimination between transcript-derived cDNA and residual gDNA background. With the addition of negative controls.

Fig. S 2 – BSG-A Dissociation Curve. BT Peak at approximately 85 °C and NC2 peak at approximately 89 °C.

Fig. S 3 – BSG-B Dissociation Curve. BT Peak at approximately 85 °C and NC2 peak at approximately 89 °C.

Fig. S 4 - BSG-C Dissociation Curve. BT Peak at approximately 85 °C and NC2 peak at approximately 89 °C.

Fig. S 5 - BSG-D Dissociation Curve. BT Peak at approximately 85 °C and NC2 peak at approximately 89 °C.

Fig. S 6 - Dissociation Curve for controls run on genomic DNA. Genomic DNA BT Peak at approximately 85 °C and Genomic DNA NC2 peak at approximately 87 °C.

# 2. Preliminary RNA Extraction Tests

To establish the final protocol, several RNA extraction strategies were systematically tested. An overview of the resulting RNA concentrations and purity ratios 260/280 and 260/230) is provided in Table S1.

- The method described by Cortés-Maldonado et al., (2020) was found to be unsuitable for both pure mycelium and BSG-D, primarily due to the physical difficulty of passing BSG-containing lysates through a 26-gauge needle.
- **Method B** (RNeasy Mini Kit) achieved high purity but demonstrated inconsistent yield across biological replicates (Table S1; Fig. S7).
- **Methods C and D** (Phenol/chloroform extraction, with and without DNase treatment, respectively) yielded sufficient material, though the ribosomal RNA band intensity in Method D was notably lower than in Method E (Trizol coupled with RNeasy column purification).
- **Method E** provided the highest overall RNA purity and structural integrity for single samples (Fig. S8); this performance prompted the integration of a laboratory-made RNA stabilization buffer to improve sampling consistency.
- **Method F** (Pure Trizol protocol) yielded acceptable chemical purity but resulted in significant RNA degradation (Fig. S8).
- **Method G** (Testing the protocol used in this study without the intermediate centrifugation step to remove most of RNA stabilization buffer) led to significantly diminished RNA purity.
- **Method H** (Application of the final protocol to side-stream residues following *Rhizopus oryzae* fermentation and alkaline protein extraction from Rusbjerg-Weberskov et al., (2025)) was evaluated to assess protocol robustness in extreme pH environments. It was observed that the highly alkaline pH of these substrates heavily influenced extraction success. Supplementation with MgSO_4_ during fermentation (Method H).

Table S 1 - Overview of preliminary tested RNA extraction methods and resulting concentration, 260/280 and 260/230 ratios. A Reference number is shown in the last column, which corresponds to the numbering in Fig S 7 and Fig S 8.

| Method | Description/Reference | Biomass | Conc. [ng/µL] | 260/280 | 260/230 | Gel Ref. |
| --- | --- | --- | --- | --- | --- | --- |
| A | Cortés-Maldonado et al., 2020 | Mycelium | 102 | 1.84 | 0.1 | - |
| A | Cortés-Maldonado et al., 2020 | Mycelium | 82 | 1.8 | 0.08 | - |
| A | Cortés-Maldonado et al., 2020 | BSG-D | 22 | 1.78 | 0.28 | - |
| A | Cortés-Maldonado et al., 2020 | BSG-D | 11.5 | 1.91 | 0.19 | - |
| B | RNeasy Mini Kit | BSG-D | 885 | 2.21 | 1.98 | Fig. S7 |
| B | RNeasy Mini Kit | BSG-D | 977 | 2.21 | 2.15 | Fig. S7 |
| B | RNeasy Mini Kit | BSG-D | 847 | 2.21 | 1.53 | Fig. S7 |
| B | RNeasy Mini Kit | BSG-D | 1623 | 2.23 | 2.34 | Fig. S7 |
| C | Phenol/chloroform | BSG-D | 303 | 2.19 | 1.34 | Fig. S8 |
| C | Phenol/chloroform | BSG-D | 391 | 2.19 | 2.02 | Fig. S8 |
| D | Phenol/chloroform (No DNase) | BSG-D | 342 | 2.22 | 2.26 | Fig. S8 |
| D | Phenol/chloroform (No DNase) | BSG-D | 320 | 2.19 | 2.22 | Fig. S8 |
| E | Trizol + RNeasy column | BSG-D | 379 | 2.22 | 2.3 | Fig. S8 |
| E | Trizol + RNeasy column | BSG-D | 248 | 2.19 | 1.11 | Fig. S8 |
| F | Trizol | BSG-D | 2241 | 2.24 | 2.52 | Fig. S8 |
| F | Trizol | BSG-D | 1692 | 2.25 | 2.58 | Fig. S8 |
| G | This study – No removal of RNA buffer | Mycelium | 1646 | 1.45 | 0.43 | - |
| G | This study – No removal of RNA buffer | Mycelium | 349 | 1.73 | 0.12 | - |
| G | This study – No removal of RNA buffer | BSG-D | 122 | 1.74 | 0.43 | - |
| G | This study – No removal of RNA buffer | BSG-D | 71 | 1.65 | 1.39 | - |
| F | This study | Alkaline side-stream | 53 | 1.95 | 0.22 | - |
| F | This study | Alkaline side-stream | 18 | 1.63 | 0.3 | - |
| F | This study | Alkaline side-stream | 21 | 1.77 | 1.27 | - |
| F | This study | Alkaline side-stream | 128 | 1.8 | 0.2 | - |
| F | This study | Alkaline side-stream | 25 | 1.93 | 0.7 | - |
| F | This study | Alkaline side-stream | 17 | 1.74 | 0.83 | - |
| F | This study | Alkaline side-stream | 43 | 1.92 | 0.89 | - |
| F | This study | Alkaline side-stream | 58 | 1.82 | 1.22 | - |
| F | This study | Alkaline side-stream | 32 | 1.92 | 0.53 | - |
| H | This study (+ MgSO4) | Alkaline side-stream | 29 | 1.77 | 1.27 | - |
| H | This study (+ MgSO4) | Alkaline side-stream | 36 | 2.1 | 1.89 | - |
| H | This study (+ MgSO4) | Alkaline side-stream | 40 | 2.1 | 1.52 | - |
| H | This study (+ MgSO4) | Alkaline side-stream | 15 | 1.8 | 0.31 | - |
| H | This study (+ MgSO4) | Alkaline side-stream | 37 | 2.03 | 0.82 | - |
| H | This study (+ MgSO4) | Alkaline side-stream | 37 | 2.08 | 1.48 | - |

Fig. S 7 – Method B

Fig. S 8 – Method C, D, E, F

Intense bands at 200 bp in lane 2 are the positive controls with genomic DNA, Lane 1 is a cDNA sample contaminated with gDNA (before doing DNase treatment), where the band at 200 bp is NC2 with exons and introns whereas the band at 100 bp is just the exons, Lane 3 is the negative control (Fig. s9).

Fig. S 9 – gDNA contamination

# References

Cortés-Maldonado, L., Marcial-Quino, J., Gómez-Manzo, S., Fierro, F., & Tomasini, A. (2020). A method for the extraction of high quality fungal RNA suitable for RNA-seq. *Journal of Microbiological Methods*, *170*, 105855. https://doi.org/10.1016/j.mimet.2020.105855

Rusbjerg-Weberskov, C. E., Foley, J. D., Yang, L., Terp, M., Gregersen Echers, S., Orlien, V., & Lübeck, M. (2025). Combined Rhizopus oryzae Fermentation and Protein Extraction of Brewer’s Spent Grain Improves Protein Functionality. *Food and Bioprocess Technology*. https://doi.org/10.1007/s11947-025-03989-1
